# Enhanced biosurfactant production through cloning of three genes and role of esterase in biosurfactant release

**DOI:** 10.1186/1475-2859-10-49

**Published:** 2011-06-27

**Authors:** Kamaljeet Kaur Sekhon, Sunil Khanna, Swaranjit Singh Cameotra

**Affiliations:** 1 Department of Biotechnology and Environmental Sciences, Thapar University, Patiala - 147001, Punjab, India; 2 NIIT University, Neemrana, Rajasthan - 301705, India; 3 Institute of Microbial technology, Sector 39-A, Chandigarh - 160036, India

## Abstract

**Background:**

Biosurfactants have been reported to utilize a number of immiscible substrates and thereby facilitate the biodegradation of panoply of polyaromatic hydrocarbons. Olive oil is one such carbon source which has been explored by many researchers. However, studying the concomitant production of biosurfactant and esterase enzyme in the presence of olive oil in the *Bacillus *species and its recombinants is a relatively novel approach.

**Results:**

*Bacillus *species isolated from endosulfan sprayed cashew plantation soil was cultivated on a number of hydrophobic substrates. Olive oil was found to be the best inducer of biosurfactant activity. The protein associated with the release of the biosurfactant was found to be an esterase. There was a twofold increase in the biosurfactant and esterase activities after the successful cloning of the biosurfactant genes from *Bacillus subtilis *SK320 into *E.coli. *Multiple sequence alignment showed regions of similarity and conserved sequences between biosurfactant and esterase genes, further confirming the symbiotic correlation between the two. Biosurfactants produced by *Bacillus subtilis *SK320 and recombinant strains *BioS a, BioS b, BioS c *were found to be effective emulsifiers, reducing the surface tension of water from 72 dynes/cm to as low as 30.7 dynes/cm.

**Conclusion:**

The attributes of enhanced biosurfactant and esterase production by hyper-producing recombinant strains have many utilities from industrial viewpoint. This study for the first time has shown a possible association between biosurfactant production and esterase activity in any *Bacillus *species. Biosurfactant-esterase complex has been found to have powerful emulsification properties, which shows promising bioremediation, hydrocarbon biodegradation and pharmaceutical applications.

## Background

Biosurfactants are surface active agents of microbial origin. They have the unique property of lowering the interfacial tension between two liquids. Biosurfactants act on the interface and are amphipathic molecules with both hydrophilic and hydrophobic moieties present within the same molecule. In literature the terms 'biosurfactants' and 'bioemulsifiers' are often used interchangeably owing to their unusual emulsifying property that makes them the molecules that enhance the accessibility and bioavailability of hydrophobic chemicals by forming stable emulsions and lowering the surface tension.

Economy is often the drawback of all biotechnological processes, especially in the case of biosurfactant production. The economics of producing biosurfactants has limited its commercial applications [1], but the production cost can be reduced by improving yield, rate, recovery and using cheap or waste substrates.

Biosurfactants have carved a niche for themselves with their unusual antibacterial, antifungal and antiviral activities [2,3]. In biomedical sciences, some of the uses of biosurfactants include their role as anti-adhesive agents to pathogens, making them useful for treating many diseases and as therapeutic, probiotic and pharmaceutical agents [1,2,4,5]. In addition biosurfactants have a huge repertoire that enables them to degrade a wide range of organic pollutants [6]. The prospects of biosurfactants have a great potential because of their applications in the petroleum industry [1,7,8] and microbial enhanced oil recovery [9-14]. The *Rhodococcus ruber *biosurfactants are found to be 1.4 to 2.3 times more efficient then the synthetic surfactants (Tween 20, Tween 60) in enhanced crude oil desorption and mobilization from soil core, with 65-82% crude oil recovery [15].

There is increasing interest for isolating new enzymes and new enzyme producing strains for their use in industrial conversions [16]. Among these enzymes lipases, esterases, cellulases, xylanases and pectinases play an important role. Esterases have proven to be versatile enzymes as they catalyze reverse reactions, namely ester synthesis and transesterification (in non-aqueous systems), and they can catalyze stereoselective and regioselective reactions, making them good candidates for the production of optically active compounds used in the pharmaceutical and agricultural industries. Moreover, esterases and lipases show activity on a great variety of substrates, with no requirement for added cofactors [17]. Thus, they are very interesting biocatalysts for industrial purposes such as detergency, flavour production, paper recycling, chemical synthesis and resolution of racemic mixtures [18].

The present paper will focus on biosurfactant yield enhancement through cloning and the role played by esterases in the production of biosurfactants. The correlation between biosurfactant production and esterase activity in *Bacillus *species has not been explored in the literature so far. The aim of the study is to investigate the range of renewable and non-conventional substrates that can be used for enhanced biosurfactant production, making it a commercially viable process. The recombinant strains need to be exploited and the technology transfer to biotechnology industries can be extremely beneficial.

## Materials and methods

### Bacterial strains, vectors and growth conditions

*Bacillus subtilis *(designated as *Bacillus subtilis *SK320 throughout the paper) was isolated from endosulfan (organochlorine pesticide) sprayed cashew plantation soil in Ernakulum, Kerala (India). The isolate was identified as *Bacillus subtilis *by MTCC, Institute of Microbial Technology, Chandigarh, India. *E. coli *DH5α was also obtained from MTCC. pGEM-T easy vector system was obtained from Promega Corporation, Madison, USA and was used according to the manufacturers instructions. *Bacillus subtilis *SK320 and the three recombinants were grown at 37°C, 120 rpm in the basal media Bushnell Hass Broth (BHB) in a 100 ml flask with 0.5% olive oil (v/v) (Olio di Oliva, Sasso, Milano, Italy) as a carbon source.

### Biosurfactant and/or Bioemulsifying Activity

Bioemulsifying activity of the biosurfactant was measured with the culture supernatant obtained by centrifuging the bacterial growth at 12,000 × *g *at 4°C for 30 min. Later 5 ml of supernatant in a glass tube and 100 μl of mobile oil was added and the contents were vortexed vigorously for 1 min at full speed and then left undisturbed for 10 min [19]. Bioemulsifying activity of the biosurfactant was measured at 550 nm spectrophotometrically (U-2001, Hitachi) in glass cuvette against blank of un-inoculated medium (5 ml) with 100 μl of mobile oil vortexed similar to the sample. Mobile oil or 2T Engine oil (Racer 2, Hindustan Petroleum Corporation Ltd, Govt. of India Enterprise, Mumbai) was obtained from a local petrol station. It is recommended as a pre-mix and oil injection system for 2 stroke engines.

### Surface tension measurement

The reduction in surface tension (dynes/cm) was measured by the ring method (Du-Nouy principle) using a tensiometer (Petro-Diesel Instruments Company, Jencon, Calcutta, India). The purified biosurfactant from *Bacillus subtilis *SK320 and the recombinants were dissolved at a concentration of 1 mg/100 ml i.e. CMC of 10 mg/L in distilled water and used for surface tension measurements against distilled water as a control.

### Esterase Activity

Esterase was measured using 100 mM para-nitrophenyl (pNp) acetate as substrate and 75 mM phosphate buffer containing 10 mM MgSO_4 _(pH 7.0). After 30 min of incubation at 37°C enzyme activity was monitored spectrophotometrically (U-2001, Hitachi) by measuring the increase in optical density at 405 nm. Specific activity was expressed as μmoles/mg protein/min [20].

### Cloning of the biosurfactant genes

Chromosomal DNA from *Bacillus subtilis *SK320 was isolated using the Rose method [21] and amplified using gene specific primers (Table 1). The primers were designed using the following website: http://frodo.wi.mit.edu/primer3. The primers were prepared by Operon Biotechnologies, Nattermannallee, Germany and supplied by Genetix Biotech Asia Private Ltd, New Delhi, India. The reaction mixture containing the chromosomal DNA, reverse primer, forward primer, dNTP mix, Taq DNA polymerase, Taq buffer and sterile water was amplified using GeneAmp PCR System 9700 (Applied Biosystems, Foster, CA, USA), with the program set to denaturation at 94°C for 5 min, annealing at 45°C for 1 min and extension at 72°C for 1 min for a total of 30 cycles, with a final extension at 72°C for 10 min. The PCR amplified product of the chromosomal DNA was ligated and cloned into the pGEM-T easy vector system. Transformation was carried out by CaCl_2 _method [22]. The transformants were selected on Amp^+ ^X-gal IPTG plates. The nucleotide sequences of the genes viz. *sfp, sfp0 *and *srfA *were determined by the dideoxy-chain termination method [23] using the Applied Biosystems DNA sequencer.

**Table 1:** Gene specific primers used for amplification of chromosomal DNA of Bacillus subtilis SK320

Gene	Primer Sequence 5'-3'	Recombinants (designation)
***sfp***	5'-CGTTCGCTCAGTCATAAGCA-3'	***BioS a***
	5'-CCTGTATGCACACCCATCTG-3'	
***sfp0***	5'-CTAGAATTCAGATTTACGGAATTTATATG-3'	***BioS b***
	5'-GGGGAATTCAGGGTGTGCGGCGCATAC--3'	
***srfA***	5'-TCCGTTTTTCCTTGTTCACC-3'	***BioS c***
	5'-TCTTTCTGCCACTGCATCAC-3'	

### Determination of the expression of biosurfactant genes

For testing the expression of the biosurfactant genes, *Bacillus subtilis *SK320 and the positive transformants (recombinants) viz.*BioS a, BioS b *and *BioS c *were grown on a basal medium containing 0.5% (v/v) olive oil at 37°C, respectively. Ampicillin was supplemented in the medium used for growing the positive transformants. Growth, biosurfactant and esterase activities were estimated at an interval of 24 h for 5 days, respectively.

### Recovery and purification of the biosurfactant

*Bacillus subtilis *SK320 and the recombinants were grown in BHB containing 0.5% olive oil (v/v) as the carbon source. After 48 h, when the biosurfactant activity was observed to be maximum in the supernatant, the culture was harvested at 7000 × *g *for 30 min at 4°C. Supernatant was filtered through Whatman No. 42 filter paper and 3 volumes of chilled acetone was added to the supernatant and left at -20°C. After 18-24 h the solution was centrifuged at 7000 × *g *for 30 min at 4°C, air dried and then dissolved in water. This step was repeated 2-3 times for purification. The final precipitate was dissolved in water and the sample was lyophilized (Heto LyoLab 3000 Lyophilizer, Germany). The lyophilized sample was then estimated for biosurfactant activity at 550 nm. For estimating total biosurfactant recovery the purified (lyophilized) powder was weighed and reported as g/L.

### Characterization of the biosurfactant

#### Lipid

The dried partially purified sample was extracted with chloroform: methanol solution (2:1, v/v). The suspension was allowed to stand at room temperature for 5-10 min and then centrifuged and supernatant collected. An aliquot (0.2 volume) of distilled water was added to the sample so as to remove any traces of chloroform:methanol, if present. The sample was then vortexed. After giving 2-3 washings with MQ water, the lower layer containing the lipid was transferred to a fresh tube and the sample was lyophilized and weighed to get the total lipid content.

#### Ash

A known weight of lyophilized sample was taken in a pre-weighed glass crucible and kept in the oven for 1 h at 80°C. After evaporating the excessive moisture the charred sample was transferred to the silica crucible and weighed. The sample was then kept in an electric muffle furnace at 550 ± 50°C for 5 h. The cooled crucible was then weighed for residual ash.

#### Protein

Extracellular protein in the supernatant and protein content in the purified biosurfactant was measured at 310 nm using the biuret method [24] using bovine serum albumin as standard. The total volume of protein sample was made up to 2 ml with Milli-Q water and 1 ml of biuret reagent. After 10 min incubation at room temperature, the absorbance was measured at 310 nm against a reagent blank in a spectrophotometer (U-2001, Hitachi).

#### Carbohydrate

The carbohydrate content was measured at 620 nm using the anthrone method [25] using glucose as standard. The total volume was made to 2 ml with sample, distilled water and 2 ml of anthrone reagent (0.2 g anthrone in 100 ml conc. H_2_SO_4_). The test tubes were covered and kept in a boiling water bath. After 10 min, the test tubes were cooled down by incubation on ice for 5 min to stop the reaction. Absorbance was read at 620 nm against a reagent blank.

### Effect of Proteinase K and Lipase (Steapsin) on purified biosurfactant

Proteinase K and Lipase were procured from MBI Fermentas and Himedia, respectively.

#### Proteinase K

The purified biosurfactant (1 mg/ml) was incubated with different concentrations of Proteinase K (viz. 0.5, 1, 2, 4 mg) at 37°C. Samples were withdrawn at different time intervals (i.e. 10, 30, 60, 120 mins) and assayed for biosurfactant activity at 550 nm.

#### Lipase (Steapsin)

The purified biosurfactant was incubated with 100, 200, 500, 700 and 1000 μg of lipase and incubated at 37°C. Samples were withdrawn at regular intervals (i.e. 30, 60, 90, 120 mins) and assayed for biosurfactant activity at 550 nm.

## Results and Discussion

### Regulation of Biosurfactant production with various substrates

*Bacillus subtilis *SK320 was grown on basal medium containing various substrates (0.5%, v/v) and monitored for growth and biosurfactant activity. The various carbon sources used were vegetable oil, glycerol, maltose, n-dodecane, mobile oil, crude oil, olive oil, glucose and sucrose. *Bacillus subtilis *SK320 was also grown on tween 40, tween 60 and triton X-100, but as these three substrates are also effective synthetic surfactants, formation of emulsion (foam) in the medium at 37°C was observed with increasing incubation time, this made it difficult to analyse the growth. Among the various carbon sources tested for biosurfactant production by *Bacillus subtilis *SK320 in basal medium olive oil, glucose, glycerol and sucrose, produced maximum activity in the range of 0.859 to 0.121 (optical density). Low levels of biosurfactant activity in the range of 0.074 to 0.014 (optical density) were detected when grown in the presence of maltose, vegetable oil, mobile oil, n-dodecane, crude oil (Table 2). During the years wide range of carbon sources such as peat hydrolysate for *B. subtilis *[26], soy bean curd residue (okara) for *B. subtilis *YB8 and *B. subtilis *MI113 [27], n-hexadecane, paraffinic oil, babassu oil for *P. aeruginosa *PA1 [28], and soybean oil, safflower oil, glycerol for *P. aeruginosa *GS9-119 and DS10-129 [29], glycerol [30], molasses medium supplemented with soya-okra for *P. aeruginosa *MTCC 2297 [31] have been reported to induce biosurfactant production. Cheap substrates such as vegetable oils and oil wastes, plant-derived oils, lactic whey and distillery wastes, starchy substrates, olive oil mill effluent, animal fat, soapstock and molasses have the potential for enhancing biosurfactant production [32]. The oils mostly used for biosurfactant production are edible oils and are not cheap [33]. The novelty and viability of the biosurfactant production process depends equally on the yield and the rate of production. Therefore, the oils used for biosurfactant production should not be very expensive but should be economical enough to give the industry a cost-effective technology.

**Table 2:** Biosurfactant activity, esterase activity, extracellular protein and biosurfactant yield of Bacillus subtilis SK320 on various substrates after 72 h

Substrate	Biosurfactant activity (OD at 55 nm)	Esterase activity (IU/ml)	Extracellular Protein (micrograms/ml)	Biosurfactant Recovery (g/L)
Vegetable oil	0.048	0.4248	2.66	0.15
Glycerol	0.137	0.6616	2.37	0.21
Maltose	0.074	0.7929	1.23	0.18
n-dodecane	0.019	0.1338	0.32	0.08
Mobile oil	0.035	0.1853	0.18	0.09
Crude oil	0.014	0.1081	0.23	0.06
Olive oil	0.859	4.382	6.97	1.2
Glucose	0.185	0.3552	2.37	0.27
Sucrose	0.121	0.5097	2.29	0.19

† Results represented as mean of at least three replicate experiments

The biosurfactant activity in the supernatant, containing olive oil as substrate, increased with time. Maximum activity was achieved during the stationary phase between 48 to 72 h of growth when the nutrient limiting conditions started prevailing in the growth medium. The production of surfactin in culture broth of *Bacillus subtilis *[34], rhamnolipids by *Pseudomonas aeruginosa *[35], emulsan in *Acinetobacter calcoaceticus *RAG-1 [36], exopolysaccharide in *A. calcoaceticus *BD4 [37] and rhamnolipid AP-6 in *P. fluorescens *378 [38] were all found to be growth associated. The rate of growth of all these bacteria varied but maximum biosurfactant activity was only observed when the bacterium entered the stationary phase irrespective of its growth rate. It was postulated that in bacteria, growth and product formation (biosurfactant) proceed as separate events [39]. *Bacillus subtilis *SK320 grew luxuriantly in the basal medium and was also capable of emulsifying olive oil to a greater extent. The low level of biosurfactant activity at 120 h coincided with the complete emulsification of olive oil in the basal medium, visualized as a milky appearing growth medium. Similar results showed that the bioemulsifier emulsan produced by oil-degrading microorganism *Acinetobacter venetianus *RAG-1 [36] and the biosurfactant produced by *Pseudomonas aeruginosa *[40] was responsible for forming stable oil-water emulsions with hydrophobic substrates such as hexadecane. The extracellular protein analyzed was maximum (6.97 mg/ml) when olive oil was used as the substrate followed by vegetable oil, glycerol and glucose. The protein content was found to be minimum with mobile oil and crude oil with values as low as 0.18 and 0.23 mg/ml.

### Induction of esterase activity

Esterase activity was found to be correlated to biosurfactant production in *Bacillus subtilis *SK320. Olive oil was the best inducer of esterase activity amongst all the substrates with the highest activity of 4.382 IU/ml, followed by maltose, glycerol, sucrose and vegetable oil with activities of 0.7929, 0.6616, 0.5097 and 0.4248 IU/ml, respectively (Table 2). This is in contrast to the observation in *Pseudomonas fluorescens *DSM 50106, *Rhodosporidium toruloides, Pseudomonas putida *NRRL B-18435 and *Acinetobacter calcoaceticus *RAG-1 where maximum esterase activity was observed in a nutrient rich medium with glucose as the carbon source [20,41-43]. But it was shown that production of type I and II esterases by *Bacillus licheniformis *S-86 was substantially enhanced by 1.6 and 2.2 times, when oils and surfactants were supplied as carbon sources [44]. The majority of the esterase produced in *Bacillus subtilis *SK320 was extracellular as the cells, after sonication, gave very low esterase activity. In *Acenitobacter *sp. [45], most of the esterase activity was found to be cell associated and only slight amounts appeared in the external medium during late growth whereas in *A. calcoaceticus *RAG-1 [46] esterase activity was found both in cell free broth and on the cell surface. With a decrease in the cell-bound activity there was an increase in the cell free esterase activity during growth. In *Bacillus subtilis *SK320 the production of esterase increased progressively during growth following the same trend as that of the biosurfactant production and was maximum at 72 h. After 72 h there was a reduction in the esterase activity after which it remained constant until 192 h. In *A calcoaceticus *BD 413 [47] a large amount of esterase and biosurfactant activity was produced during the transition from the exponential to the stationary phase, while in *A calcoaceticus *RAG-1 [36] esterase activity followed the growth pattern, with the maximum activity being achieved during the stationary phase of growth. In *Acinetobacter venetianus *RAG-1 the release of emulsan from the bacterial cell surface was mediated by the action of a cell surface esterase, which is one of the key components in the active emulsan-protein complex and itself appears in the growth medium just prior to the appearance of the cell-free emulsifying activity [47].

### Cloning and expression of biosurfactant genes

*Bacillus subtilis *SK320 and recombinant strains *BioS a, BioS b *and *BioS c*, cultivated on a basal medium containing 0.5% (v/v) olive oil at 37°C were compared for their growth, biosurfactant and esterase activities. Growth was observed to be maximum in *Bacillus subtilis *SK320 followed by *BioS a, BioS b *and *BioS c*, whereas biosurfactant and esterase activities were found to be enhanced in the recombinants in comparison to the parent strain (Table 3). There was negligible growth in the case of *E.coli *DH5α strain when it was grown on a basal medium containing olive oil indicating that *E.coli *DH5α was unable to utilize olive oil as the carbon source. The screening of biosurfactant and esterase positive strains was also confirmed from the fact that *E.coli *strains DH5α, HB101 and MM294 could not grow on simple triglycerides [43]. There was no apparent biosurfactant and esterase activity found in the parent *E.coli *DH5α, whereas the recombinant strains which possess an active biosurfactant gene were now able to utilize olive oil as a carbon source. Data revealed that the biosurfactant genes were not only successfully cloned and expressed but they were over-expressed in *BioS a, BioS b *and *BioS c *showing a twofold increase in the activity then the parent strain (Table 3). The biosurfactant activity increased in the first 24 h producing the maximum activity between 48 to 72 h. After 72 h biosurfactant activity started showing a decline reaching a minima at 120 h. Maximum biosurfactant activity was observed in case of the recombinant *BioS b *followed by *BioS a *and *Bio c*. Olive oil supplemented in the culture media was found to be completely emulsified after 120 h. Cloning of the biosurfactant genes from *Bacillus subtilis *SK320 into *E.coli *not only resulted in the expression of the biosurfactant activity but also conferred esterase production in the recombinant cells. Similarly, cloning, sequencing and characterization of a genomic region of *B. subtilis *B3 comprising *srfDB3, aspB3, lpab3 *and *yczEB3 *genes was carried out and it was observed that the *srfDB3 *gene encodes thioesterase which is required for biosynthesis of surfactin in *B. subtilis *[48]. Esterase activity of *BioS a, BioS b *and *BioS c *was found to be enhanced when compared to *Bacillus subtilis *SK320.

**Table 3:** Growth, biosurfactant and esterase activity of Bacillus subtilis SK320 and recombinants at 72 h of growth

Source	Biosurfactant activity (OD 550 nm)	Esterase activity (IU ml^-1^)	Growth (OD 600 nm)
*Bacillus subtilis *SK320	0.859	4.382	0.514
*BioS a*	1.354	8.293	0.351
*BioS b*	1.259	8.521	0.296
*BioS c*	1.198	8.465	0.489
*E. coli *DH5α	0.157	0.101	0.132

† Results represented as mean of at least three replicate experiments

### Phylogenetic analysis of three biosurfactant genes

**Figure 1:**
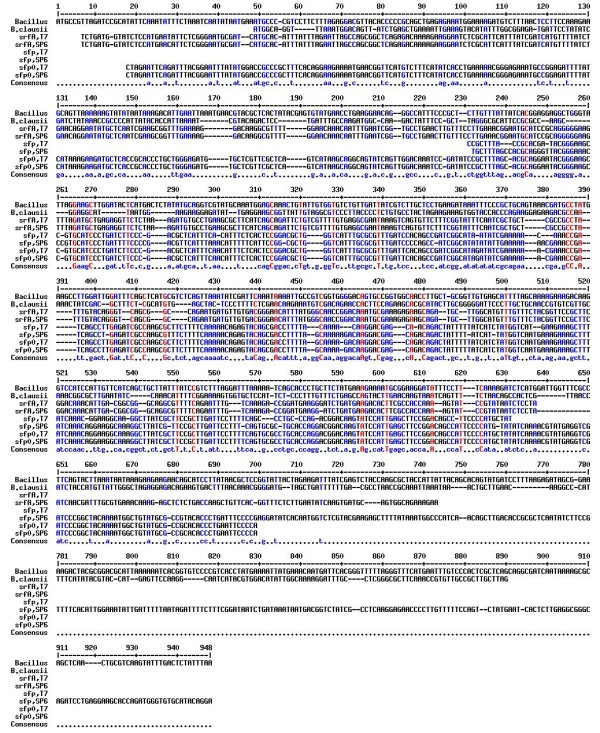
**MultAlin (Multiple Sequence Alignment) of sfp, sfp0 and srfA gene sequences with esterase gene sequences from NCBI database**. The consensus sequences are shown in the last line of the alignment table. Bacillus = Bacillus sp. NK13 esterase gene (Liu et al. 2005, PubMed Accession number: DQ196347). B. clausii = Bacillus clausii KSM-K16 esterase gene (Hakamada et al. 1994, PubMed Accession number: AP006627). sfp, sfp0 and srfA are gene sequences responsible for biosurfactant production in Bacillus subtilis SK320.

The genes *sfp *(1210 bp), *sfp0 *(642 bp) and *srfA *(707 bp) were successfully cloned and sequenced using an Applied Biosystems DNA Sequencer (DDBJ/EMBL/GeneBank accession numbers are: EU822921, EU822922 & EU822923). The DNA sequences were used to infer functional and evolutionary relationships between sequences in the database and to identify members of gene families using the NCBI BLAST (http://www.ncbi.nlm.nih.gov/BLAST) facility. The results revealed sequence homology of the *sfp, sfp0 *and *srfA *genes with *Bacillus subtilis *surfactin synthetase gene, *Bacillus amyloliquefaciens*, *Bacillus licheniformis*, *Bacillus megaterium, Bacillus subtilis *srfAA, several lipases from yeast and filamentous fungi to name a few. In addition much weaker similarities were also observed. Based on the matching sequences found with BLAST, it was estimated that the greatest overall similarity (99.0%) was with the biosurfactant and esterase genes of *Bacillus subtilis. *Multiple alignments of the deduced amino acid sequences from *sfp, sfp0 *and *srfA *were carried out with two esterase gene sequences viz. *Bacillus *sp. NK13 esterase gene (Accession number: DQ196347) [Liu G, Tan Z, Zhang J. Cloning of esterase gene from *Bacillus *sp. NK13. Submitted] and *Bacillus clausii *KSM-K16 esterase gene (Accession number: AP006627) [49]. The results indicated similarity and conserved family characteristics between the biosurfactant and esterases genes, confirming our prediction of a possible correlation between the two activities (Figure 1).

### Purification and characterization of Biosurfactant

*Bacillus subtilis *SK320 and the recombinants *BioS a, BioS b *and *BioS c*, were grown in a basal medium with 0.5% (v/v) olive oil and the purified biosurfactant was lyophilized to obtain a clear powder. The biosurfactant yield (%) obtained from *BioS a, BioS b *and *BioS c *was much higher than that obtained for parent *Bacillus subtilis *SK320 i.e. 1.2 g/L (Table 4). Surface tension values were found to be 72.1 dynes/cm for tap water and 70.7 dynes/cm for sterile Milli-Q water. Biosurfactant extracted from the parent *Bacillus subtilis *SK320 was able to reduce the surface tension of tap water to 40.1 dynes/cm, whereas the biosurfactant obtained from recombinants *BioS a, BioS b *and *BioS c *reduced the surface tension of tap water to as low as 38.4, 35 and 30.7 dynes/cm, respectively (Table 4). Results suggested that the successful expression of the biosurfactant gene was responsible for the surface tension reduction by the biosurfactants. Total production yield of the lipopeptides from *Bacillus subtilis *BBK-1 was about 480 mg/L at 30°C for 24 h [50], whereas *Bacillus *sp. strain IAF 343 gave the yield of 1 g/L on medium containing only water soluble substrates [19]. *P. aeruginosa *DS10-129 produced 4.31, 2.98 and 1.77 g/L rhamnolipid biosurfactant using soybean oil, safflower oil and glycerol as substrates [51]. *Bacillus cereus *IAF 346 produced a monoglyceride biosurfactant that lowered the surface tension of water to 28 mN/m with a yield of 1.6 g/L (pH 6.5) and 1.7 g/L (pH 7.0) [19]. *B. subtilis *grown on medium containing 4% glucose gave the yield of 1-2 g/L of biosurfactant with minimum surface tension of 27 mN/m [52]. *B. licheniformis *JF-2 anaerobically produced biosurfactant when grown in glucose rich medium and reduced surface tension of water to 28 mN/m [53]. Purified lichenysin A from *B. licheniformis *BAS50 decreased the surface tension of water to 28 mN/m with a yield of 70-160 mg/L [54]. The maximum yield of surfactin was approximately 110 mg/lit by the strain *B. subtilis *S 499 [55]. In a very recent study, biosurfactant production of 1.74 g/L was observed when the microbial consortium of *Enterobacter cloacae *and *Pseudomonas *sp. (ERCPPI-2) was grown on minimal salt medium supplemented with olive oil (1%, w/v) and 1% (w/v) sodium nitrate supplemented with 1.39% (w/v) K_2_HPO_4 _at 40°C and 150 rpm after 48 h incubation. The consortium ERCPPI-2 was able to reduce the surface and interfacial tensions to 31.7 and 0.65 mN/m [14].

**Table 4:** Surface tension values and yield of purified biosurfactant from Bacillus subtilis SK320 and its recombinants

	Biosurfactant recovery (g/L)	Surface tension (dynes/cm)
*Bacillus subtilis *SK320	1.2	40.1
*BioS a*	2.13	38.4
*BioS b*	2.20	35
*BioS c*	2.45	30.7

† Results represented as mean of at least three replicate experiments

Various biochemical and physiological properties of the biosurfactants were studied after its purification. The biochemical analysis revealed that the purified biosurfactant from *BioS a *had 89.70% lipid, 14.9% ash, 7.21% protein and 3.08% carbohydrate content. Biosurfactant from *BioS b *contained 90.30% lipid, 15.20% ash, 6.73% protein and 2.94% carbohydrate content whereas, biosurfactant obtained from *BioS c *contained 91.0% lipid, 15.38% ash, 6.26% protein and 2.70% carbohydrate content, respectively. The biosurfactant from all the three recombinant strains had a high lipid content thereby affirming that the biosurfactants belong to the class of lipopeptides. These results were in accordance with *Bacillus subtilis *SK320 biosurfactant, which had 7.45% protein, 89.40% lipid, 3.15% carbohydrate and 14.5% ash content, respectively.

**Figure 2:**
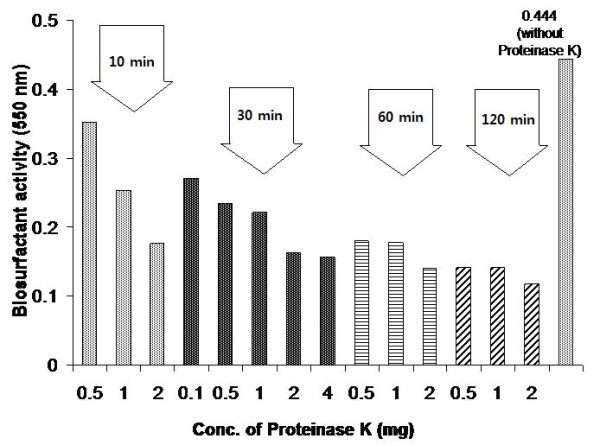
**Effect of Proteinase K on purified biosurfactant**. The purified biosurfactant was incubated at 37°C with various proteinase K concentrations for 10, 30, 60 and 120 min. The sample was analyzed for biosurfactant activity at 550 nm.

**Figure 3:**
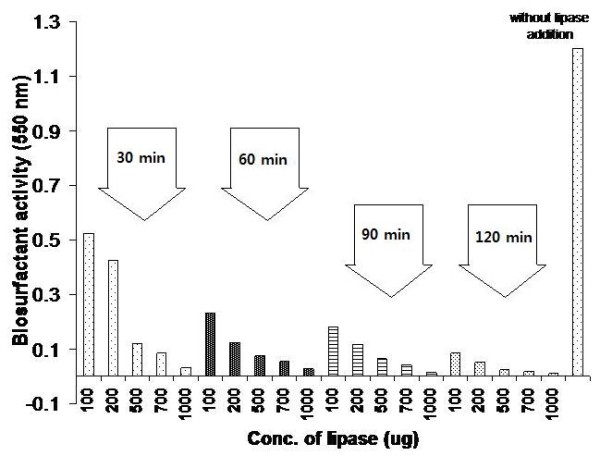
**Effect of lipase on purified biosurfactant**. The purified biosurfactant was incubated at 37°C with various lipase concentrations for 30, 60, 90 and 120 min. The sample was analyzed for biosurfactant activity at 550 nm.

Boiling or incubating the biosurfactant with proteinase K led to a decrease in biosurfactant and emulsifying activity, indicating that the protein (esterase) moiety was essential for biosurfactant activity (Figure 2). The decrease in activity of the purified biosurfactant at pH 8.0 and above also indicated an active role of the protein moiety, as the loss in activity at high pH may be due to the denaturation of the protein. Lipids constituted a large percentage of the purified biosurfactant and therefore regulated the biosurfactant activity in a significant manner. The incubation of biosurfactant with lipase resulted in appreciable loss in biosurfactant activity (up to 100%) (Figure 3). In *Pseudomonas *PGI [56], the protein moiety was shown to be essential for biosurfactant activity as incubation of the biosurfactant with chymotrypsin reduced both solubilizing and emulsifying activities to very low levels. Similarly, in *A. calcoaceticus *BD4, the polysaccharide moiety of the biosurfactant alone showed no emulsification activity, however polysaccharide released with protein during growth showed potent emulsification activity [57]. In *A. calcoaceticus *RAG-1, the protein moiety of the emulsan was not at all involved in the activity as the deproteinized emulsan retained all its biosurfactant activity. Emulsan was later characterised to be a lipopolysaccharide [46]. The biosurfactant of *Bacillus subtilis *SK320 contained 7.45% protein (w/w), which was almost one third when compared to 34% (w/w) in *Pseudomonas *PG-1 [56]. The amount of lipid was more when compared with the lipid content (32%, w/w) in *Pseudomonas *PG-1. The difference in the chemical composition could be due to the different growth conditions. *Bacillus subtilis *SK320 was grown on basal medium with olive oil as the carbon source, while *Pseudomonas *PG-1 was grown in minimal medium with hexadecane/pristane as the substrate [46,58].

## Conclusions

*Bacillus subtilis *SK320 produces a biosurfactant that belongs to the class of lipopeptides having excellent emulsifying properties. It has been shown that biosurfactant production is mediated by extracellular esterase in the growth medium of *Bacillus subtilis *SK320 and its recombinant strains. Biosurfactants devoid of protein or lipid components showed much lower emulsifying activity. The recombinant strains showed a twofold increase in the esterase activity and biosurfactant yield. The biosurfactants were capable of reducing the surface tension of water to a significantly lower value. These properties are of great importance in bioremediation and various other industrial applications.

## Competing interests

The authors declare that they have no competing interests.

## Authors' contributions

KSS has carried out the research. SK has supervised the study and SSC assisted with his stimulating discussions and revision of the manuscript. All authors have read and approved the final manuscript.
