# Erratum to: Enhanced biosurfactant production through cloning of three genes and role of esterase in biosurfactant release

**DOI:** 10.1186/s12934-017-0761-2

**Published:** 2017-08-22

**Authors:** Kamaljeet Kaur Sekhon, Sunil Khanna, Swaranjit Singh Cameotra

**Affiliations:** 1 Department of Biotechnology and Environmental Sciences, Thapar University, Patiala, Punjab 147001 India; 2 NIIT University, Neemrana, Rajasthan 301705 India; 3 104, 10A, Chandigarh, 160036 India

## Erratum to: Microbial Cell Factories 2011, 10:49 DOI 10.1186/1475-2859-10-49

The authors wish to publish an erratum to address concerns that were raised by one of the readers of their article [1].

Post publication peer review by the journal found that surfactin or esterase production could not have occurred as described in the article because the recombinant *Escherichia coli* strains do not possess all the DNA sequences required for their production. Moreover, the regions that were used for the nucleotide sequence alignment of biosurfactant biosynthesis and esterase genes do not show any significant amino acid sequence similarity, which undermines the conclusions about the homology of these genes.

The authors provided the following explanation:

1. The authors do not claim that the biosurfactants produced by *Bacillus subtilis* SK320 and its recombinants are “surfactin”—the biosurfactants reported here belong to the class of lipopeptides having excellent emulsifying properties as stated clearly in “Conclusions” section. If any statement in the article is implying that the biosurfactants produced by *Bacillus subtilis* SK320 are “surfactin”, it should be considered as an error.
2. The primers used to amplify the *Bacillus subtilis* SK320 genomic DNA are named or designated randomly. It should not be understood from the designation that only a particular gene or gene sequence was targeted or amplified using that particular primer. As it is an error made accidently, the authors would like to rename the primers as Pa, Pb and Pc instead of sfp, sfp0 and srfA (Table 1). The article does not suggest anywhere that the biosurfactant production can happen only in the presence of one gene, it requires the entire operon and so was the objective of each primer.

**Table 1:** Gene specific primers used for amplification of chromosomal DNA of *Bacillus subtilis* SK320

Primers	Primer sequence 5ʹ-3ʹ	Recombinants (designation)
Pa	5ʹ-CGTTCGCTCAGTCATAAGCA-3ʹ	BioS a
	5ʹ-CCTGTATGCACACCCATCTG-3ʹ	
Pb	5ʹ-CTAGAATTCAGATTTACGGAATTTATATG-3ʹ	BioS b
	5ʹ-GGGGAATTCAGGGTGTGCGGCGCATAC-3ʹ	
Pc	5ʹ-TCCGTTTTTCCTTGTTCACC-3ʹ	BioS c
	5ʹ-TCTTTCTGCCACTGCATCAC-3ʹ
3. The authors would like to emphasize that “There was no apparent biosurfactant and esterase activity found in the parent *E. coli* DH5α strain, whereas the recombinant strains were able to utilize olive oil as a carbon source” (statement cited from section: “Cloning and expression of biosurfactant genes” under “Results and Discussion”). Since the recombinant *E. coli* strains were able to utilize olive oil as a carbon source, it was the first step towards claiming the successful cloning of the biosurfactant genes.
4. The authors encourage the readers to refer to the GenBank accession numbers of the gene sequences submitted to NCBI’s nucleotide database given in the article and make their own alignments and do comparisons for studying the homology of these genes.
